# Communication interventions for medically unexplained symptom conditions in general practice: A systematic review and meta-analysis of randomised controlled trials

**DOI:** 10.1371/journal.pone.0277538

**Published:** 2022-11-14

**Authors:** Ailish Katherine Byrne, Arabella Scantlebury, Katherine Jones, Laura Doherty, David J. Torgerson

**Affiliations:** 1 Department of Health Sciences, York Trials Unit, University of York, Heslington, York, United Kingdom; 2 Warwick Clinical Trials Unit, University of Warwick, Coventry, United Kingdom; Amsterdam UMC Locatie VUmc, UNITED STATES

## Abstract

**Background:**

Medically unexplained symptoms (MUS) account for 3–50% of all General Practitioner (GP) consultations and are difficult to diagnose due to their unknown aetiology, symptom overlap between conditions, and lack of effective treatment options. MUS patients’ and primary care clinicians frequently face challenges during consultations, with GPs reporting difficulty identifying and classifying MUS, whilst patients report stigma and feeling illegitimised by clinicians. Communication interventions have been proposed as a method to facilitate the doctor-patient relationship and aid the management of MUS.

**Aim:**

This systematic review aims to evaluate the effectiveness of primary care based communication interventions at improving MUS patients’ and/or clinician outcomes.

**Method:**

Four electronic databases were searched from inception to November 2021. Two researchers independently undertook screening, data extraction and quality appraisal. Given the heterogeneous nature of the studies identified, narrative syntheses were conducted, along with meta-analyses where possible to pool data.

**Results:**

9 papers from 10 Randomised Controlled Trials were included. The included studies displayed considerable risk of bias and poor reporting. Some limited evidence suggests that communication interventions tailored to MUS and not following a pre-specified model (such as reattribution) could improve pain, mental and physical functioning whilst reattribution training may improve clinician confidence treating MUS. However, methodological limitations mean that these findings should be interpreted with caution.

**Conclusion:**

A range of interventions for improving communication with MUS patients in primary care have been evaluated. However, the heterogeneous nature of existing evidence and poor study quality mean we cannot conclude whether these interventions are effective. Before considering further randomised controlled trials researchers should focus on developing a new or modified communication intervention for MUS patients and their clinicians.

**Trail registration:**

The systematic review was prospectively registered with PROSPERO (registration record CRD42020206437).

## Introduction

Medically Unexplained Symptoms (MUS conditions) are estimated to account for between 3–50% of all general practice consultations globally [1,2]. Defined as “physical symptoms with no identified organic cause” lasting for at least three months [3,4], MUS conditions are difficult to diagnose due to their seeming lack of organic cause, symptom overlap and unknown aetiology [5]. Patients suffering from MUS conditions tend to experience psychological distress, social isolation, and reduced quality of life (QOL). This has been estimated to cost the UK over £17 billion, of which £3 billion are NHS costs [4,6].

The Royal College of General Practitioners [7] emphasised the necessity of managing patients’ symptoms and focusing on the doctor-patient relationship in order to reduce the number of unnecessary and long investigations. Recommendations included the need to connect with the patient, summarise tangible explanations, and provide reassurance.

One way to achieve this is through enhanced communication and collaborative care (e.g.: integrating physical therapy, pain clinic and psychological care services). Previous research has suggested that communication interventions may alleviate the difficulties experienced by clinicians in the treatment and management of MUS conditions [8].

Despite research highlighting the importance of the doctor-patient relationship for MUS patients through promoting resilience, effective illness management, and helping maintain QOL [9,10], difficulties between MUS patients and their clinicians are still frequently seen within primary care. Communication interventions may provide a practical, achievable way to improve the doctor-patient relationship. This systematic review aimed to evaluate the effectiveness of primary care based communication interventions for patients with MUS and clinicians. More specifically, we aimed to explore:

1. Is there evidence that communication interventions within primary care have an impact on patient outcomes?
2. Is there evidence that communication interventions within primary care have an effect on clinician confidence and attitudes towards MUS patients?
3. What are the key components of current communication interventions?

## Method

The systematic review was prospectively registered with PROSPERO (registration record CRD42020206437) and followed the Preferred Reporting Items for Systematic Reviews and Meta-Analyses (PRISMA) guidelines [11].

### Selection criteria

Studies were assessed against the eligibility criteria described in Table 1.

**Table 1:** PICOS eligibility criteria.

	Inclusion criteria	Exclusion criteria
Population/participants	Primary care doctors and nurses. MUS patients, 16 years and older, either clinically or self-diagnosed with an MUS condition.	Doctors or nurses working in a non-primary care setting e.g. secondary care, voluntary sector, or other medical specialists such as physiotherapists, rheumatologists and medical students. MUS patients who have not received a formal diagnosis and those under the age of 16 years.
Intervention	Training interventions (as defined by study authors) aimed at improving primary care professionals’ communication skills when consulting with MUS patients. Training interventions included but were not restricted to: reattribution, patient-centered care, cognitive behavioral skills and shared decision-making. Training could be delivered by any method, including face-to-face and virtually.	Communication interventions delivered to patients.
Comparator	No training, usual care or a comparative intervention.	
Outcomes	We were interested in patient and clinician outcomes relevant to change in patient care (e.g.: perceived improvement in patient-doctor relationship or improved patient outcomes). In terms of patient outcomes, we were interested in pain, mental functioning, physical functioning, depression, anxiety, somatization, quality of life, and satisfaction with care. Clinician outcomes of interest were satisfaction with care, beliefs/attitudes towards MUS patients, perception of the doctor-patient relationship, and perceived usefulness of training.	Lack of useable data
Study design	Randomised Controlled Trials (RCTs).	All other study designs.

### Search strategy

The search strategy was developed by the authors in collaboration with a Liaison Librarian at the University of York. Four databases were searched from inception to November 2021 for RCTs published in English: Embase, CENTRAL, MEDLINE and CINAHL. An example search strategy is available in S8 Table. The reference and citation lists of all papers that reached full-text screening, were screened for eligibility regardless of whether they were included within the final review.

### Study selection and data extraction

At all stages of screening (title, abstract and full-text), papers were reviewed by two researchers (AB and LD or KJ) independently using the systematic review software Rayyan, with disagreements resolved through discussion with a third researcher, where necessary (LD or KJ). Throughout study selection, an inclusion/exclusion checklist was used by all reviewers to promote consistency. A data extraction form was created using Google Forms and piloted by three researchers (AB, LD and KJ). The data extracted included: participant characteristics, methodology, context, interventions and outcome data. To assist comparisons between studies, mean, standard deviation and interquartile ranges were extracted to allow calculation of mean differences and 95% confidence intervals, where these were not already provided. Data extraction was conducted independently by three researchers (AB and LD or KJ), with any disagreements resolved as a group.

### Risk of bias

Risk of bias (RoB) was assessed independently by two reviewers (AB and KJ) using the revised Cochrane Risk of Bias Tool (RoB 2.0) or the Cochrane RoB 2.0 for cluster-randomised trials. Discrepancies were resolved through discussion or by deferring to a third researcher as required.

### Data analysis

First, we present a narrative and tabular summary of key study characteristics of the included studies. We then describe the available evidence under two categories: patient outcomes and clinician outcomes. Narrative and tabular summaries are used to present data for each outcome measure of interest at baseline and the final follow-up point. Statistical outcomes are provided as reported by the trial in the first instance, with standard deviation, mean difference and standardised mean difference being calculated if not provided, using Cochrane Review Manager Version 5. For instances in which standard deviations were calculated by author AB the calculations have been provided in S1 Table.

Due to the substantial heterogeneity of interventions and outcome measures across studies, meta-analysis was possible for two outcomes—anxiety and depression. For these outcomes, meta-analyses were undertaken using Cochrane Review Manger Version 5 and recommendations from the Cochrane Handbook were adhered to account for the design effect [12]. If statistical heterogeneity was noted (I^2^>40%), a random-effects model was used to account for expected heterogeneity between studies. The below equation was used to reduce each trial down to its ‘effective sample size’ [13]:

1+(M‐1)xICC

The intracluster correlation coefficients (ICC) at 12 months were estimated using the baseline ICC provided by Schaefert et al. [14]. Raw data on the cluster and sample size was extracted from each trial to populate the ‘M’ of the equation. As the ICCs provided by Schaefert et al. [14] were at baseline, the design effect calculations were also based upon baseline numbers of clusters and participants. Design effect calculations are available in S2 Table.

## Results

**Fig 1:**
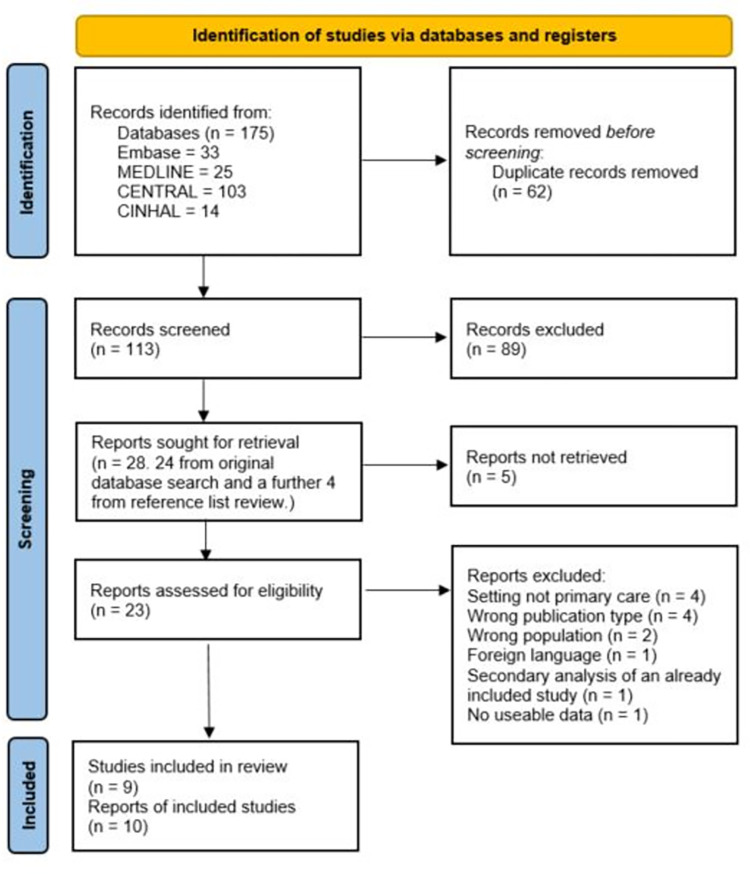
PRISMA flow diagram illustrating the flow of studies through the review process.

After deduplication, we screened 113 records and included nine RCTs reported in 10 articles (Fig 1). A list of excluded studies is available in S3 Table.

### Study characteristics

Nine RCTs were conducted between 2001 and 2012 in: Germany [14–16], Spain [17,18], UK [19,20], and Denmark [21–23]. Studies recruited GPs/family physicians as their primary care population. Only one study [20] also included Nurse Practitioners. All trials adopted a cluster design, with one [16] also being a cross-over trial. An overview of the study characteristics is provided in Table 2.

**Table 2:** Study characteristics.

Study	Study Design	Number of primary care clinicians randomised		Number of participants recruited				Medical condition	Risk of bias
		Intervention	Control	Intervention	Control	Intervention	Comparator		
Alamo, Moral & de Torres. [18]	Cluster RCT	10	10	63	47	Patient-centered approach	Usual care	FM or GMCP	High risk
Toft et al. [23]	Cluster RCT	19	19	195	155	TERM model (reattribution)	GPs provided with definitions of somatic disorder	Somatic disorder	High risk
Schaefert et al. [14]	Cluster RCT	18	17	183	145	Enhanced medical care + collaborative group intervention	Enhanced medical care	Unclear mixture of MUS conditions	Some concern
Larisch et al. [15]	Cluster RCT	23	19	73	54	Reattribution technique	Routine psychosocial care	Unclear mixture of MUS conditions	Some concern
Morriss et al. [20]	Cluster RCT	35	35	66	75	Reattribution technique	Usual care	Unclear mixture of MUS conditions	Some concern
Rosendal et al. ^1^ [21]	Cluster RCT	23	20			TERM model of reattribution	GPs provided with definitions of somatization	Unclear mixture of MUS conditions	Some concern
Rosendal et al.^2^ [22]	Cluster RCT	20	17	506	405	TERM model of reattribution	GPs provided with definitions of somatization	Unclear mixture of MUS conditions	High risk
Aiazaguena et al. [17]	Cluster RCT	19	20	76	80	Specific communication intervention tailored for somatization	Reattribution technique	Unclear mixture of MUS conditions	Some concern
Morriss et al. [19]	Cluster RCT	35	35	-	-	Reattribution	No training	-	High risk
Rief et al. [16]	Cluster RCT with cross-over	12	14	Unclear	Unclear	“How to manage patients with unexplained physical symptoms” training package	No training	Unclear mixture of MUS conditions	High risk

- = Not reported ^1, 2 =^ two associated papers from the same trial * = Interquartile range + = 95% confidence interval FM = Fibromyalgia GMCP = generalised musculoskeletal chronic pain.

The included studies all described using a form of communication intervention as their experimental condition, with six trials using reattribution training [15,19–23], one using communication and psychosocial techniques [17], one trained GPs how to communicate with these patients [16], one employed the patient-centered approach [18], and one using collaborative training in psychosomatic illness and the management of somatizing patients [14]. An overview of the interventions evaluated by the included studies is provided in Appendix 2. There were 387 clinicians with a mean age of 47.7 years and an average 14.3 years working in primary care recruiting 2,412 patients. The patient cohort consisted of substantially more females than males, mean age over 35 years (SA 3). The majority of patients were employed and either married or living with a spouse. Recruited patients had been diagnosed with either Fibromyalgia, Chronic musculoskeletal pain or Somatic Disorder in two studies [18,23], the remaining trials included an unspecified range of MUS conditions.

### Risk of Bias (ROB)

Overall, the included studies displayed considerable risk of bias and were poorly reported. Reporting omissions relating to the randomisation or allocation process, use of intention-to-treat analysis (ITT), measurement of outcome and analysis plan were particularly common [16–19,22,23]. Three trials raised concern over baseline imbalances in recruitment [18,20,22], whilst three RCTs displayed high rates of missing data or dropouts with no description of how these were accounted for during analysis [16,22,23]. All studies displayed a potential risk of bias due to lack of blinding, and inconsistencies in time points and/or incomplete reporting of follow-up or outcome data were observed across the majority of trials. For instance, Aiarzaguena et al. [17] reported having 3-, 8- and 12 month follow-up points, but only disclosed follow-up data at 12 months. RoB decisions for each domain of the ROB2 tool are illustrated in S4 and S5 Tables.

### Outcome measures

Three studies included clinician outcome measures relevant to this review [16,19,21]: whether the intervention affected clinicians’ confidence in treating MUS, perceived usefulness of the training program, satisfaction with care, and clinicians’ beliefs regarding MUS conditions.

Eight studies assessed patient outcome measures relevant to this review [14–18,20,22,23] across eight domains including: anxiety, depression, physical functioning, pain, mental functioning, somatization score, QOL, and patient satisfaction with their clinician.

All trials had follow-up points after completion of the intervention. However due to heterogeneity across trials in relation to when these follow-ups occurred (e.g.: 4 weeks, 3, 6, 12 and 24 months), each trials’ baseline and final follow-up data only have been reported. ‘Follow-up’ has been used as the blanket heading regardless of when the final follow-up point occurred. S6 Table provides follow-up data at 3, 6, 12 & 24 months.

### Results from studies

#### Pain

Five cluster trials [17,18,20,22,23] assessed pain as an outcome measure (Table 3). These trials evaluated the utility of reattribution training [20,22,23], patient centered care [18], and a standardised communication technique tailored for somatizing patients [17] (SA 2). Two trials were removed from analysis as they only reported data at baseline [20,23]. The remaining three trials measured pain using the Short Form-36 (SF-36) bodily pain scale [17,22], Nottingham Health Profile (NHP) [18], or measures of pain intensity and pain as a problem [18]. Table 3 shows that some differences are relatively large. Aiarzaguena et al. [17], which evaluated a specific, standardised communication technique, and Rosendal et al. [22], which evaluated reattribution training for somatization, showed a statistically significant decrease in pain at 12 months.

**Table 3:** Outcome data–pain.

Author	N at follow-up	Measure	Baseline	Follow-up	Mean difference at follow-up between intervention and control [95% CI]	Standardised mean difference at follow-up	P value at follow-up
**Rosendal et al. [22]**	287 293	SF-36 Bodily pain	**Control:** 48.0 [44.8, 51.1] ^a^ 48.0 (15.89)* Intervention: 49.6 [46.8, 52.4] ^a^ 49.6 (32.11)*	**Control:** 10.5 ^b^ [8.5, 12.5] ^a^ 58.5 (17.27)* Intervention: 6.5 ^b^ [3.8, 9.3] ^a^ 56.1 (23.96)*	-2.40 [-5.79, 0.99]	-0.11 [-0.28, 0.05]	.020
**Alamo, Moral & de Torres. [18]**	33 48 33 48 33 48	Pain intensity Pain as a problem NHP-pain	**Control:** 6.8 (1.9) **Intervention:** 6.9 (1.8) **Control:** 4.1 (0.8) **Intervention:** 3.4 (1.2) **Control:** 53.3 (32.1) **Intervention:** 49.2 (30.5)	**Control:** 6.6 (2.1) **Intervention:** 5.9 (2.6) **Control:** 3.9 (0.8) **Intervention:** 3.1 (1.0) **Control:** 52.7 (28.3) **Intervention:** 42.3 (34.4)	-0.70 [-1,73, 0.33] -0.80 [-1.19, -0.41] -10.40 [-24.11, 3.31]	-0.29 [-0.73, 0.16] -0.86 [-1.32, -0.39] -0.32 [-0.77, 012]	.14 .73 .08
**Aiarzaguena et al. [17]**	74 72	SF-36 Bodily pain	**Control:** 46.2 (25.2) **Intervention:** 43.6 (24.4)	**Control:** 1.72 ^b^ [-2.69, 6.13] ^a^ 47.92 (19.35)* Intervention: 11.41 ^b^ [6.98, 15.85] ^a^ 55.01 (19.16)*	7.09 [0.85, 13.33]	0.37 [0.04, 0.69]	.003

Mean and standard deviation unless specified otherwise. Items left blank were not provided within the paper.

a = 95% CI.

b = Mean difference.

* = mean and/or standard deviation calculated by AB.

#### Mental functioning

Five cluster trials [14,15,17,22,23] assessed mental functioning as an outcome measure. These trials evaluated the utility of reattribution training [15,22,23], a specific communication intervention for somatisation [17], and enhanced collaborative psychodynamic training [14]. One paper [23] only reported data at baseline and was removed from analysis. The remaining four trials reported mental functioning using the SF-36 [14,17,22] or SF-12 [15]. For all outcomes a positive effect was indicative of improved functioning. Table 4 shows that some differences are relatively large.

**Table 4:** Outcome data–mental functioning.

Author	N at follow-up	Measure	Baseline	Follow-up	Mean difference at follow-up between intervention and control [95% CI]	Standardised mean difference at follow-up [95% CI]	P value at follow-up
**Rosendal et al. [22]**	283 299 247 245	SF-36 Mental Health Subscale SF-36 Mental component summary	**Control:** 67.0 [64.4, 69.5]^a^ 67.0 (21.86)* Intervention: 65.6 [63.4, 67.8] ^a^ 65.6 (19.36)* Control: 47.4 [45.8, 49.0] ^a^ 47.4 (12.89) **Intervention:** 41.4 [44.9, 47.9] ^a^ 41.4 (12.05)	**Control:** 1.0 ^b^ [-1.9, 3.8] ^a^ 68 (24.38)* Intervention: 0.4 ^b^ [-1.9, 2.6] ^a^ 66 (19.88)* Control: 0.2 ^b^ [-1.4, 1.8] ^a^ 47.6 (12.83)* **Intervention:** -0.6 ^b^ [-1.8,0.6]^a^ 40.8 (9.55)*	-2.00 {-5.63, 1.63] -6.8 [-8.80,- 4.80]	-0.09 [-0.25, 0.07] -0.60 [-0.78, -0.42]	.760 .420
**Larisch et al. [15]**	34 44	SF-12 Mental	**Control:** 41.0 (10.3) **Intervention:** 37.6 (9.6)	**Control:** 4.3 ^b^ (3.6) 45.3* (3.6) **Intervention:** 2.2 ^b^ (5.2) 39.8*(5.2)	-5.50 [-7.46, -3.54]	-1.19 [-1.68, -0.70]	.479
**Schaefert et al. [14]**	108 143	SF-36 Mental component summary	**Control:** 40.38 (11.42) **Intervention:** 41.55 (10.16)	**Control:** 42.09 (11.77) **Intervention:** 46.59 (10.76)	4.50 [1.66, 7.34]	0.40 [0.15, 0.65]	.022
**Aiarzaguena et al. [17]**	74 72	SF-36 Mental Health Subscale	**Control:** 50.3 (20.2) **Intervention:** 50.1 (21.6)	**Control:** 5.63^b^ [2.50, 8.76]^a^ 55.93 (13.67)* Intervention: 10.27^b^ [7.12, 13.42]^a^ 60.37 (13.65)*	4.44 [0.01, 8.87]	0.32 [-0.00, 0.65]	.063

Mean and standard deviation unless specified otherwise. Items left blank were not provided within the paper.

a = 95% CI.

b = Mean difference.

* = mean and/or standard deviation calculated by AB.

However, only one study (Schaefert et al. [14]) which evaluated a collaborative group intervention with enhanced care showed a statistically significant improvement in mental functioning at 12 months.

#### Somatization

Five cluster trials [14–16,22,23] assessed somatization score as an outcome measure. These trials assessed the utility of reattribution training [15,22,23], communication training [16], and enhanced, collaborative psychodynamic care [14]. One trial [23] only reported data at baseline and was therefore removed from analysis. The remaining four trials measured somatization using the Symptom Checklist—Somatisation (SCL-SOM) [22], Somatoform Symptoms-7 (SOMS-7) [15], Somatic Symptom Interview (SSI) [16], Somatic Symptom Count (SOMS symptom count) [16], or Patient Health Questionnaire-15 (PHQ-15) [14]. For all measures a negative score was indicative of milder somatic symptoms. Outcome data is provided in Table 5. None of the studies showed a statistically significant reduction in somatization at 12 months.

**Table 5:** Outcome data–somatization.

Author	N at follow-up	Measure	Baseline	Follow-up	Mean difference at follow-up between intervention and control [95% CI]	Standardised mean difference at follow-up [95% CI]	P value at follow-up
**Rosendal et al. [22]**	284 304	SCL-SOM	**Control:** 2.3^c^ (2.0–2.6)^d^ **Intervention:** 2.3^c^ (1.9–2.6)^d^	**Control:** -0.2 ^b^ [-0.3, -0.2] ^a^ **Intervention:** -0.2 ^b^ [-0.2, -0.1] ^a^			.230
**Larisch et al. [15]**	34 44	SOMS-7	**Control:** 12.3 (9.8) **Intervention:** 14.8 (8.3)	**Control:** 1.6 ^b^ (0.6) 13.9* (0.6) **Intervention:** -0.7 ^b^ (3.1) 14.1* (3.1)	0.20 [-0.74, 1.14]	0.08 [-0.36, 0.53]	.192
**Schaefert et al. [14]**	113 149	PHQ-15	**Control:** 12.66 (4.89) **Intervention:** 12.56 (4.73)	**Control:** 10.57 (5.10) **Intervention:** 9.55 (5.12)	-1.02 [-2.27, 0.23]	-0.20 [-0.44, 0.05]	.079
**Rief et al. [16]**	114 85 114 85	SSI SOMS symptom count	**Control:** 6.8 (3.8) **Intervention:** 8.0 (4.1) **Control:** 13.8 (8.2) **Intervention:** 15.5 (8.2)	**Control:** 5.9 (4.1) **Intervention:** 5.9 (4.2) **Control:** 13.7 (8.2) **Intervention:** 15.3 (9.5)	0.00 [-1.17, 1.17] 1.60 [-0.92, 4.12]	0.00 [-0.28, 0.28] 0.18 [-0.10. 0.46]	

Mean and standard deviation unless specified otherwise. Items left blank were not provided within the paper.

a = 95% CI.

b = Mean difference.

c = median.

d = 25th– 75th percentiles.

* = mean and/or standard deviation calculated by AB.

#### Anxiety

Eight cluster trials [14–18,20,22,23] reported anxiety as an outcome measure. These trials evaluated the utility of reattribution training [15,20,22,23], patient-centered care [18], an enhanced, collaborative interpersonal training [14], communication training [16], and a standardised communication technique for somatization [17]. One trial [17] only provided data at baseline and therefore cannot provide any comparison information. The remaining seven trials measured anxiety using the Symptom Checklist-8 (SCL-8) [22,23], Hospital Anxiety and Depression Scale—Anxiety (HADS-A) [15], Whiteley-7 [14,23], Whitely Anxiety Index (WAI) [16], Beck Anxiety Inventory [16], General Health Questionnaire–Anxiety (GHQ-anxiety) [18], or by assessing cases in terms of number and percentage [20]. For all measures a higher score corresponded to worse anxiety. Table 6 shows that some differences are relatively small; only Alamo, Moral and de Torres [18] which evaluated patient-centered care showed a statistically significant reduction in anxiety at 12 months. Whilst Toft et al. [23] reported a statistically significant p-value at 24 months (p = .001), they didn’t provide the raw data to support this.

**Table 6:** Outcome data–anxiety.

Author	N at follow-up	Measure	Baseline	Follow-up	Mean difference at follow-up between intervention and control [95% CI]	Standardised mean difference at follow-up [95% CI]	P value at follow-up
**Rosendal et al. [22]**	285 306	SCL-8	**Control:** 1.9^c^ (1.5–2.6)^d^ **Intervention:** 2.0^c^ (1.5–2.8)^d^	**Control:** -0.2 [-0.3, -0.1]^a^ **Intervention:** -0.1 [-0.2, 0.0]^a^			.320
**Larisch et al. [15]**	34 44	HADS-A	**Control:** 7.7 (4.2) **Intervention:** 9.3 (3.9)	**Control:** -0.3 ^b^ (0.7) 7.4* (0.7) **Intervention:** -0.8^b^ (0.7) 8.5* (0.7)	1.10 [0.79, 1.41]	1.56 [1.04, 2.07]	.419
**Schaefert et al. [14]**	113 149	Whiteley-7	**Control:** 10.37 (6.24) **Intervention:** 10.86 (6.61)	**Control:** 8.57 (6.93) **Intervention:** 7.66 (6.60)	-0.91 [-2.57, 0.75]	-0.13 [-0.38, 0.11]	.061
**Morriss et al. [20]**	75 66	Caseness (n) and %	**Control:** 46 (61%) **Intervention:** 40 (64%)	**Control:** 27 (36%) **Intervention:** 31 (47%)			.101
**Toft et al. [23]**	125 154	SCL-8 Whiteley-7	**Control:** 16^c^ (12–21)^d^ **Intervention:** 16^c^ (12–21)^d^ **Control:** 13^c^ (10–17)^d^ **Intervention:** 13^c^ (10–17)^d^		3.0 [0.8, 3.9]^x^		.001
**Alamo, Moral & de Torres [18]**	33 48	GHQ-anxiety	**Control:** 5.2 (2.9) **Intervention:** 6.2 (2.4)	**Control:** 5.4 (2.8) **Intervention:** 4.6 (4.8)	-0.80 [-2.46, 0.86]	-0.19 [-0.64, 0.25]	.040
**Rief et al. [16]**	114 85 114 85	BAI WI	**Control:** 11.8 (10.0) **Intervention:** 14.4 (10.3) **Control:** 5.4 (3.1) **Intervention:** 6.2 (2.9)	**Control:** 11.5 (9.2) **Intervention:** 11.8 (10.6) **Control:** 4.6 (3.1) **Intervention:** 5.0 (3.3)	0.30 [-2.52, 3.12] 0.40 [-0.50, 1.30]	0.03 [-0.25, 0.31] 0.13 [-0.16, 0.41]	

Mean and standard deviation unless specified otherwise. Items left blank were not provided within the paper.

a = 95% CI.

b = Mean difference.

c = median.

d = 25th– 75th percentiles.

* = mean and/or standard deviation calculated by AB.

x = mean difference provided within original data.

#### Depression

Eight cluster trials [14–18,20,22,23] assessed the effect of a communication intervention upon patient’s depression score compared to a control. These trials evaluated the utility of reattribution training [15,20,22,23], patient-centered care [18], communication training [16], an enhanced, collaborative interpersonal training [14], and a standardised communication technique for somatization [17]. One trial [17] only provided baseline data and therefore cannot provide any comparison information. The remaining seven trials measured depression using the SCL-8 [22,23], HADS-D [15], PHQ-9 [14]. Beck Depression Inventory (BDI) [16], GHQ-depression [18], or cases expressed in number and percentage [20]. For all measures a higher score indicated poorer mental health. Outcome data is provided in Table 7. One trial [23] did not provide any outcome data, concluding that ‘no significant difference was found’ for depression. None of the included studies showed a statistically significant reduction in depression at 12 months.

**Table 7:** Outcome data–depression.

Author	N at follow-up	Measure	Baseline	Follow-up	Mean difference at follow-up between intervention and control [95% CI]	Standardised mean difference at follow-up [95% CI]	P value at follow-up
**Rosendal et al. [22]**	285 306	SCL-8	**Control:** 1.9^c^ (1.5–2.6)^d^ **Intervention:** 2.0^c^ (1.5–2.8) ^d^	**Control:** -0.2^b^ [-0.3, -0.1]^a^ **Intervention:** -0.1^b^ [-0.2, 0.0] ^a^			.320
**Larisch et al. [15]**	34 44	HADS-D	**Control:** 6.3 (3.5) **Intervention:** 7.9 (4.5)	**Control:** **-**0.2 ^b^ (0.7) 6.1 (0.7)* Intervention: -0.8 ^b^ (0.0) 7.1 (0.0)*	1.00 [0.76, 1.24]	2.15 [1.58, 2.71]	.467
**Schaefert et al. [14]**	112 149	PHQ-9	**Control:** 9.76 (5.54) **Intervention:** 8.89 (5.11)	**Control:** 7.98 (5.25) **Intervention:** 6.29 (4.58)	-1.69 [-2.91, -0.47]	-0.35 [-0.59, -0.10]	.111
**Morriss et al. [20]**	75 66	Caseness and %	**Control:** 46 (61%) **Intervention:** 40 (64%)	**Control:** 21 (28%) **Intervention:** 18 (27%)			.873
**Toft et al. [23]**	125 154	SCL-8	**Control:** 16^c^ (12–21)^d^ **Intervention:** 16^c^ (12–21)^d^				
**Alamo, Moral & de Torres [18]**	33 48	GHQ- depression	**Control:** 3.6 (2.5) **Intervention:** 3.7 (2.5)	**Control:** 4.0 (2.1) **Intervention:** 3.2 (2.6)	-0.80 [-1.83, 0.23]	-0.33 [-0.78, 0.12]	.330
**Rief et al. [16]**	114 85	BDI	**Control:** 12.5 (8.4) **Intervention:** 13.9 (9.1)	**Control:** 11.8 (8.1) **Intervention:** 11.8 (9.5)	0.00 [- 2.51, 2.51]	0.00 [-0.28, 0.28]	

Mean and standard deviation unless specified otherwise. Items left blank were not provided within the paper.

a = 95% CI.

b = Mean difference.

c = median.

d = 25th– 75th percentiles.

* = mean and/or standard deviation calculated by AB.

#### Physical functioning

Seven cluster trials [14–18,20,22,23] assessed the effect of a communication intervention upon patient’s physical functioning compared with a control (Table 8). These trials evaluated the utility of reattribution training [15,20,22,23], patient-centered care [18], an enhanced, collaborative interpersonal training [14], and a standardised communication technique for somatization [17]. One trial [20] reported data only at baseline and has been removed from analysis. The remaining six trials assessed physical functioning as an outcome measure using the SF-36 [14,17,22,23], or NHP-physical mobility scale [18]. For all three measures a greater score is indicative of better physical functioning. Table 8 shows that differences varied widely; however, only one study (Aiarzaguena et al. [17]) which evaluated a specific, standardised communication technique for somatization was statistically significant and generated a large mean difference.

**Table 8:** Outcome data–physical functioning.

Author	N at follow-up	Measure	Baseline	Follow-up	Mean difference at follow-up between intervention and control [95% CI]	Standardised mean difference at follow-up [95% CI]	P value at follow-up
**Rosendal et al. [22]**	284 288	SF-36	**Control:** 84.2^c^ (65.0–95.0)^d^ **Intervention:** 80.0 ^c^ (61.1–95.0) ^d^	**Control:** 0.8^b^ [-0.9, 2.6]^a^ **Intervention:** 0.5^b^ [-1.7, 2.8]^a^			.890
**Larisch et al. [15]**	34 44	SF-12	**Control:** 43.0 (11.0) **Intervention:** 41.4 (8.2)	**Control:** 0.5 (-0.7)_ 43.5 (-0.7)* Intervention: 3.8 (2.0) 45.2 (2.0)*	1.70 [1.06, 2.34]	-3.08 [-3.75, -2.41]	.069
**Aiaraguena et al. [17]**	74 72	SF-36	**Control:** 70.5 (25.1) **Intervention:** 73.2 (23.2)	**Control:** 2.56^a^ (1.15, 3.97)^b^ 73.06 (6.20)* Intervention: 5.23^b^ (3.8–6.67)^a^ 78.43 (6.20)*	5.37 [3.36, 7.38]	0.86 [0.52, 1.20]	.012
**Schaefert et al. [14]**	108 143	SF-36	**Control:** 42.05 (8.88) **Intervention:** 43.16 (9.09)	**Control:** 44.14 (9.68) **Intervention:** 44.56 (9.61)	0.42 [-1.99, 2.83]	0.04 [-0.21, 0.29]	.674
**Toft et al. [23]**	111 138	SF-36	**Control:** 90.0^c^ (75.0–100.0)^d^ **Intervention:** 85.0^c^ (60.0–95.0)^d^				
**Alamo, Moral & de Torres [18]**	33 48	NHP-physical mobility	**Control:** 29.2 (19.4) **Intervention:** 22.7 (17.9)	**Control:** 32.2 (21.5) **Intervention:** 20.1 (16.3)	-12.10 [-20.76, -3.44]	**-**0.64 [-1.10, -0.19]	.100

Mean and standard deviation unless specified otherwise. Items left blank were not provided within the paper.

a = 95% CI.

b = Mean difference.

c = median.

d = 25th– 75th percentiles.

* = mean and/or standard deviation calculated by AB.

#### QOL and satisfaction with clinician

One study [20] listed QOL (reported as an overall Euroqol-5D (EQ-5D score) and ‘satisfaction with clinician’ as patient outcomes of interest. Outcome data was only reported at the 3-month follow-up period. No information on patients’ baseline QOL or satisfaction was provided, meaning that it was not possible to assess any change in participant data. The difference in QOL between intervention and control participants at 3 months failed to reach significance (95% CI [-0.40, 1.73], p = .221).

Patient satisfaction with care was reported at 3 months using number and percentages. At 3 months, 50 intervention patients (76%) reported being satisfied with the care they received opposed to 48 (64%) control patients who received usual care. The difference between groups was not statistically significant (95% CI [0.86, 14.47], p = .080).

#### Clinician outcome data

Three RCTs assessed clinician outcome measures relevant to this review [16,19,21]. Two trials investigated the effect of reattribution training and included ‘confidence treating MUS patients’ as a clinician outcome measure [19,21]. Other outcomes included ‘satisfaction with care given’, ‘MUS beliefs’ and ‘perceived usefulness of training’. One trial [16] conducted general communication training as part of their intervention, and asked GPs to respond to the question “How relevant was the workshop for your everyday practice in your GP office?”

However, reporting of clinician outcome data in all trials was limited. One trial [19], only reported clinician outcomes immediately after training, not baseline, and no outcome data was provided for control clinicians. The second trial [16] only reported on the relevance and quality of the training program (87% agreed it was at least highly relevant). No baseline or follow-up data was provided on whether the training affected their practice, knowledge or confidence. The final trial [21] used a two-part self-report questionnaire to assess clinician’s views on ‘patients with somatoform disorder’ and ‘somatizing patients’. No overall score was provided for this questionnaire, so individual items investigating confidence and beliefs of clinicians towards MUS patients were extracted. Reattribution training resulted in increased clinician confidence towards MUS patients: item 3 “I often feel unsure of what to do” (MD = -0.90 [-1.72, 0.08], p = .019) and item 16: “I feel comfortable dealing with somatizing patients” (MD = 2.5 [0.86, 4.14], p = .002). The results for beliefs towards MUS patients were more variable, as responses to item 4: “I enjoy working with these patients” indicated that only intervention clinicians found treating MUS patients to be more enjoyable (MD = 1.10 [0.27, 1.93], p = .008). No significant difference (p = .441) was found between control and intervention clinicians for item 17: “Somatization reflects a characteristic response in patients which is not amendable to change”, supported by 95% CIs that passed through zero (MD = -0.60 [-2.19, 0.99], p = .441).

### Meta-analyses

**Fig 2:**
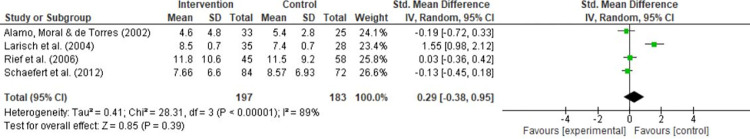
Meta-analysis using standardised, random effect to assess the effect of communication interventions upon patients’ anxiety. Data from Rief et al. [16] using the BAI is included.

**Fig 3:**
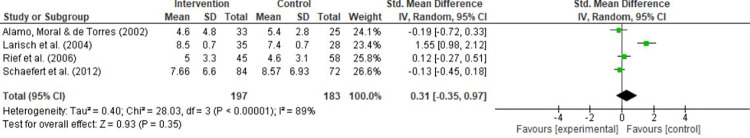
Meta-analysis using standardised, random effect to assess the effect of communication interventions upon patients’ anxiety. Data from Rief et al. [16] using the WI is included.

**Fig 4:**
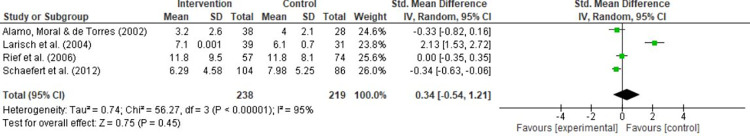
Meta-analysis using standardised, random effect to assess the effect of communication interventions upon patients’ depression.

Four RCTs that assessed anxiety and depression were suitable for meta-analysis [14–16,18]. A random effect model for continuous outcomes was chosen because of the expected clinical heterogeneity, which was confirmed statistically by high I2’s. The model was repeated for anxiety to allow for the inclusion of both anxiety outcome measures that were used by Rief et al. [16] (BAI and WI). Considerable heterogeneity was found across the four trials, (anxiety (BAI): I^2^ = 89%; anxiety (WI): I^2^ = 89%; depression: I^2^ = 95%). Meta-analysis highlighted a pooled standardised mean difference of 0.29 [-0.38, 0.95] for anxiety (BAI), 0.31 [-0.35, 0.97] for anxiety (WI), and 0.3 [-0.54, 1.21] for depression. The treatment effect did not reach statistical significance for anxiety on either meta-analysis (p = .390; p = .350) (Figs 2 and 3) or depression (p = .450) (Fig 4).

## Discussion

This systematic review identified 10 papers from 9 RCTs, which evaluated the effectiveness of primary care-based communication interventions for MUS patients. Due to a lack of high-quality evidence, we cannot draw reliable conclusions on the effectiveness of primary care based communication interventions for MUS patients and the data from the included trials should be interpreted with caution. All of the trials included within this review displayed either some or high risk of bias due to methodological flaws—including providing no information on the randomization process, no statistical analysis plan, lack of blinding, high dropout rates that were not accounted for using ITT analysis, selective reporting of data, and baseline heterogeneity. Two trials provide some evidence of a benefit to MUS patient’s pain, mental functioning, and physical functioning [14,17] and one trial [21] provided evidence that reattribution training can improve clinician’s confidence treating MUS patients. Whilst methodological flaws mean that we cannot conclude that any of the assessed communication interventions display a clear benefit to MUS patients, the potential benefit of enhanced care models such as these has been supported in other reviews. For example; van Dessel et al. [24] found that enhanced care had a comparable benefit to cognitive behavioural therapy for treating MUS. The trials using enhanced care included within this review were also found to have methodological concerns relating to blinding of participants and assessors, incomplete data sets, selective reporting, allocation concealment and treatment fidelity.

Across patient outcomes, trials that employed reattribution training were less likely to display a significant result, supporting confidence intervals, or clinically significant mean differences—supporting the previous conclusions of Gask et al. [25], whose narrative review reported that reattribution training is too simplistic to resolve the difficulties GPs face when managing MUS. The ineffectiveness of reattribution training on patient outcomes within this review may be due to their grounding in psychological explanations of MUS conditions, which are now widely contradicted [26–28], and specifically directing GPs to link patient’s physical symptoms to a psychological explanation.

By contrast, the interventions used by Schaefert et al. [14] and Aiarzaguena et al. [17] were unique, specifically tailored for MUS patients, and did not follow a pre-specified model (such as reattribution or patient-centered care). Both placed greater emphasis on the empowerment of patients, importance of legitimizing patients, and reinforcing the patients’ experience, whilst less emphasis was placed on a psychological explanation for MUS. Psychosocial and physical explanations were focused on instead [14,17]. The focus of these interventions away from psychological explanations and towards legitimizing patients could explain why these trials reported significant improvements in pain, mental functioning and physical functioning whilst reattribution interventions did not.

### Strengths and limitations

The major strengths of this review is its transparency, consistency, and clear attempts to minimize bias. Study selection, data extraction and quality appraisal were undertaken by two researchers independently using a set of agreed, standardised procedures to minimize reviewer error and bias. A meta-analysis was conducted with the support of a Senior Statistician for both outcome measures that had suitable data (anxiety and depression), and the influence of a cluster design was accounted for by calculating an adjusted sample size in both instances.

This review is limited by its use of only four databases, predominantly consisting of research published in English by Western countries. Use of more international databases, such as LILACS, would have made this review more representative. Moreover, as this review did not have the resources to translate foreign language papers, the review may have been influenced by language bias. The risk of this is deemed low as foreign language papers were only excluded at full-text screening, of which there was one (Fig 2).

Whilst a meta-analysis was conducted, the low quality of the four studies included, and the fact that each trial investigated a different communication intervention, limits how well their findings can be pooled. Intracluster correlation coefficients (ICC’s) were not provided by each of the included trials, leading to the use of the 0.09 and 0.05 baseline ICCs reported by Schaefert et al. [14] for all adjusted sample size estimates. As a result, the sample sizes may have been down weighted. A further limitation is that within the meta-analysis for anxiety, data from Schaefert et al. [14] and Rief et al. [16] was obtained from the Whitely scale, which specifically measures health anxiety opposed to general, whilst the other two included trials [15,18] used a measure of general anxiety. Health and general anxiety may be two different effects and combining these data sets may have confounded the outcome data.

### Implications and recommendations

The evidence that currently exists is of poor quality and does not support that there is a readily available communication intervention that can be implemented within primary care to benefit MUS patients or their clinicians.

The limitations of current evidence make it difficult to state exactly what direction future research should take but considering the prevalence and economic burden associated with MUS conditions this field needs to be considered a research priority. This is especially true now with the global rise of ‘long-covid’ as a medical phenomenon with considerable parallels to MUS conditions, unknown aetiology, no known treatment, and increasing prevalence worldwide.

Future research could question why the findings of this review are so variable across interventions and outcomes. Are there component parts of these interventions that work? Alternatively, based on the lack of high quality evidence available, there is an argument for developing a new intervention for primary care clinicians and MUS patients, following the Medical Research Council Guidance for developing complex interventions [29].

Further evidence syntheses are also recommended to incorporate evidence from non-randomised studies investigating communication interventions for MUS patients attending primary care and inform the development or refinement of future communication interventions for this population group. One of the challenges we faced during this review was the range of outcome measures used across the included studies, which meant that only two outcomes (anxiety and depression) were assessed by enough trials to be able to pool the data for a meta-analysis. Development of a core outcome set is therefore suggested as an additional research priority in this field.

## Conclusion

Current evidence is not robust enough to establish whether communication interventions targeted towards PCC’s have an effect on the outcomes of MUS patients or their clinicians. Our findings display some evidence that communication interventions tailored to MUS patients could benefit outcomes including pain, mental and physical functioning, and some evidence that reattribution training improves clinicians’ confidence treating these patients. However, the methodological flaws evidenced across all of the included trials mean it is not possible to conclude that a certain type of communication intervention has a definite effect. Considerable work needs to be done to establish a robust evidence base before a high quality RCT is conducted. Several possible directions for future research are proposed, including: the development of a core outcome set and the development of a new primary care-based communication intervention for MUS patients.

## Supporting information

S1 Table Standard deviation calculations. (PDF)

S2 Table Design effect sample size calculations. (PDF)

S3 Table Excluded papers spreadsheet. (XLSX)

S4 Table Risk of bias visualisation table. (PDF)

S5 Table RoB2 cluster trials quality assessment spreadsheet. (XLSX)

S6 Table Full set of outcome data tables. (PDF)

S7 Table PRISMA checklist. (PDF)

S8 Table Example search strategy. (PDF)

S9 Table Summary table of assessed communication interventions. (PDF)

S10 Table Patient characteristics table. (PDF)
