# Correction: Communication interventions for medically unexplained symptom conditions in general practice: A systematic review and meta-analysis of randomised controlled trials

**DOI:** 10.1371/journal.pone.0316850

**Published:** 2024-12-30

**Authors:** Ailish Katherine Byrne, Arabella Scantlebury, Katherine Jones, Laura Doherty, David J. Torgerson

The country listed in the Academic Editor’s affiliation is incorrect. The correct affiliation for Johannes van der Wouden is Amsterdam UMC Locatie VUmc, Netherlands.
