# Supplementary material for: Communication interventions for medically unexplained symptom conditions in general practice: A systematic review and meta-analysis of randomised controlled trials
Source: PLoS One. 2022 Nov 14;17(11):e0277538. doi: 10.1371/journal.pone.0277538 (PMC9662736; doi:10.1371/journal.pone.0277538)
Supplement: S1 Table — (PDF) [file pone.0277538.s001.pdf]

Standard deviation calculations

For studies that did not provide standard deviations, they were calculated using the following equation:

Standard error = (upper CI – lower CI)/3.92

Standard deviation = SE x √N

Pain

Rosendal et al. (2007): SF-36 Bodily Pain Scale, follow-up

Control: (12.5 – 8.5) = 4/3.92 = 1.020

√287 = 16.94 x 1.02

= 17.27

Intervention: (9.3 – 3.8) = 5.5/3.92 = 1.403

√293 = 17.11 x 1.40

= 23.95

Aiarzaguena et al. (2007): SF-36 Bodily Pain Scale, follow-up

Control: (6.13 - - 2.69) = 8.82/3.92 = 2.25

√74 = 8.60 x 2.25

= 19.35

Intervention: (15.85 – 6.98) = 8.87/3.92 = 2.26

√72 = 8.48 x 2.26

= 19.16

Mental Functioning

Rosendal et al. (2007): SF-36 Mental Health Subscale

Control, baseline: (69.5 – 64.4) = 5.1/3.92 = 1.301

√283 = 16.82 x 1.30

= 21.86

Control, follow-up: (3.8 - - 1.9) = 5.7/3.92 = 1.454

√283 = 16.82 x 1.45

= 24.38

Intervention, baseline: (67.8 – 63.4) = 4.4/3.92 = 1.122

√299 = 17.29 x 1.12

= 19.36

Intervention, follow-up: (2.6 - - 1.9) = 4.5/3.92 = 1.147

√299 = 17.29 x 1.15

= 19.88

Rosendal et al. (2007): Mental component summary

Control, baseline: (49 – 45.8) = 3.2/3.92 = 0.816

√247 = 15.72 x 0.82

= 12.89

Control, follow-up: (1.8 - - 1.4) = 3.2/3.92 = 0.82

√245 = 15.65 x 0.82

= 12.83

Intervention, baseline: (47.9 – 44.9) = 3/3.92 = 0.765

√245 = 15.65 x 0.77

= 12.05

Intervention, follow-up: (0.6 - - 1.8) = 2.4/3.92 = 0.612

√245 = 15.65 x 0.61

= 9.55

Physical functioning

Aiarzaguena et al. (2007): SF-36 Physical functioning

Control, follow up: (3.97 – 1.15) = 2.82/3.92 = 0.72

√74 = 8.6 x 0.72

= 6.20

Intervention, follow-up: (6.67 – 3.8) = 2.87/3.92 = 0.73

√72 = 8.48 x 0.73

= 6.20
