# Supplementary material for: Communication interventions for medically unexplained symptom conditions in general practice: A systematic review and meta-analysis of randomised controlled trials
Source: PLoS One. 2022 Nov 14;17(11):e0277538. doi: 10.1371/journal.pone.0277538 (PMC9662736; doi:10.1371/journal.pone.0277538)
Supplement: S2 Table — (PDF) [file pone.0277538.s002.pdf]

Supplementary material 1: adjusting for design effect – sample size calculations

Design effect and adjusted sample size calculations for anxiety at 12 months

Author	***Allocation***	***N of GPs***	***N of participants at baseline***	***Average cluster size***	***Step 1: Design effect calculation =***<br><br>***1 + (m – 1) x ICC = DE***	***Step 2: Sample size calculation***<br><br>***unadjusted sample size at 12 months/design effect***	***Step 3: Adjusted sample size****
Larisch et al. (2004)	Control	17	54	3.2	1 + ((3.2 - 1)) x 0.09) = 1.198	34/1.198	28
	Intervention	20	73	3.7	1 + ((3.7 - 1)) x 0.09) = 1.243	44/1.243	35
Schaefert et al. (2012)	Control	20	145	7.25	1 + ((7.25 - 1)) x 0.09) = 1.562	113/1.562	72
	Intervention	19	183	9.6	1 + ((9.6 - 1)) x 0.09) = 1.774	149/1.774	84
Alamo, Moral & de Torres (2002)	Control	10	47	4.7	1 + ((4.7 - 1)) x 0.09) = 1.333	33/1.333	25
	Intervention	10	63	6.3	1 + ((6.3 - 1)) x 0.09) = 1.477	48/1.477	33
Rief et al. (2006)	Control	14	166	11.9	1 + ((11.9 – 1) x 0.09) = 1.981	114/1.981	58
	Intervention	12	129	10.8	1 + ((10.8 – 1) x 0.09) = 1.882	85/1.882	45

**adjusted sample sizes are rounded to nearest whole number*
DE = Design effect

Design effect and adjusted sample size calculations for depression at 12 months

Author	***Allocation***	***N of GPs***	***N of participants at baseline***	***Average cluster size***	***Step 1: Design effect calculation =***<br><br>***1 + (m – 1) x ICC = DE***	***Step 2: Sample size calculation***<br><br>***unadjusted sample size at 12 months/design effect***	***Step 3: Adjusted sample size****
Larisch et al. (2004)	Control	17	54	3.2	1 + ((3.2 - 1)) x 0.05) = 1.11	34/1.11	31
	Intervention	20	73	3.7	1 + ((3.7 - 1)) x 0.05) = 1.135	44/1.135	39
Schaefert et al. (2012)	Control	20	145	7.25	1 + ((7.25 - 1)) x 0.05) = 1.312	113/1.312	86
	Intervention	19	183	9.6	1 + ((9.6 - 1)) x 0.05) = 1.430	149/ 1.430	104
Alamo, Moral & de Torres (2002)	Control	10	47	4.7	1 + ((4.7 - 1)) x 0.05) = 1.185	33/1.185	28
	Intervention	10	63	6.3	1 + ((6.3 - 1)) x 0.05) = 1.265	48/1.265	38
Rief et al. (2006)	Control	14	166	11.9	1 + ((11.9 – 1) x 0.05) = 1.545	114/1.545	74
	Intervention	12	129	10.8	1 + ((10.8 – 1) x 0.05) = 1.490	85/1.490	57

**adjusted sample sizes are rounded to nearest whole number*
DE = Design effect
