# Supplementary material for: Communication interventions for medically unexplained symptom conditions in general practice: A systematic review and meta-analysis of randomised controlled trials
Source: PLoS One. 2022 Nov 14;17(11):e0277538. doi: 10.1371/journal.pone.0277538 (PMC9662736; doi:10.1371/journal.pone.0277538)
Supplement: S4 Table — (PDF) [file pone.0277538.s004.pdf]

Supplementary material 3: Risk of Bias table

	RoB 2 CRT Domains							
	1a Randomisation process	***1b Timing, identification or recruitment***	***2a Deviations***	***2b Analysis***	***3 Missing outcome data***	***4 Measurement of outcome***	***5 Reported results***	**Overall risk of bias**
Rosendal et al. (2005)	+	+	-	+	+	-	-	Some concern
Rosendal et al. (2007)	+	+	-	X	X	+	-	High risk
Morriss et al. (2007)	+	-	-	+	+	-	-	Some concern
Alamo, Moral & de Torres (2002)	X	-	-	+	+	+	X	High risk
Aiarzaguena et al. (2007)	-	+	-	+	+	-	-	Some concern
Larisch et al. (2004)	+	+	-	+	+	+	-	Some concern
Schaefert et al. (2012)	+	+	-	+	+	+	-	Some concern
Toft et al. (2010)	+	+	-	X	-	-	X	High risk
Morriss et al. (2006)	-	+	-	X	X	X	X	High risk
Rief et al. (2006)	-	+	-	X	X	X	-	High risk
