# Supplementary material for: Communication interventions for medically unexplained symptom conditions in general practice: A systematic review and meta-analysis of randomised controlled trials
Source: PLoS One. 2022 Nov 14;17(11):e0277538. doi: 10.1371/journal.pone.0277538 (PMC9662736; doi:10.1371/journal.pone.0277538)
Supplement: S6 Table — (PDF) [file pone.0277538.s006.pdf]

Supplementary material 5: full outcome data

Table 1: Pain outcome data

Author	*N at 12 months*	*Measure*	*Baseline*	*3 month*	*6 month*	*12 month*	*Mean difference at 12 months between intervention and control [95% CI]*	*Standardised mean difference at 12 months*	*P value at 12 months*
Rosendal et al. (2007)	287<br><br>293	SF-36 Bodily pain	**Control:**<br>48.0 [44.8, 51.1] [a]<br>48.0 (15.89)*<br><br>**Intervention:**<br>49.6 [46.8, 52.4] [a]<br>49.6 (32.11)*	**Control:**<br>8.2 [4.6, 11.8] [a]<br>56.2 (32.36)*<br><br>**Intervention:**<br>5.0 [2.9, 7.0] [a]<br>54.6 (19.23)*		**Control:**<br>10.5 [b] [8.5, 12.5] [a]<br>58.5 (17.27)*<br><br>**Intervention:**<br>6.5 [b] [3.8, 9.3] [a]<br>56.1 (23.96)*	-2.40 [-5.79, 0.99]	-0.11 [-0.28, 0.05]	.020
Alamo, Moral & de Torres. (2002)	33<br>48	Pain intensity	**Control:**<br>6.8 (1.9)<br>**Intervention:**<br>6.9 (1.8)		**Control:**<br>6.0 (2.4)<br>**Intervention:**<br>5.7 (2.5)	**Control:**<br>6.6 (2.1)<br>**Intervention:**<br>5.9 (2.6)	-0.70 [-1.99, 0.59]	-0.29 [-0.73, 0.16]	0.14
	33<br>48	Pain as a problem	**Control:**<br>4.1 (0.8)<br>**Intervention:**<br>3.4 (1.2)		**Control:**<br>3.9 (0.8)<br>**Intervention:**<br>3.3 (1.0)	**Control:**<br>3.9 (0.8)<br>**Intervention:**<br>3.1 (1.0)	-0.80 [-1.19, -0.41]	-0.86 [-1.32, -0.39]	0.73
	33<br>48	NHP-pain	**Control:**<br>53.3 (32.1)<br>**Intervention:**<br>49.2 (30.5)		**Control:**<br>49.9 (29.3)<br>**Intervention:**<br>48.3 (33.2)	**Control:**<br>52.7 (28.3)<br>**Intervention:**<br>42.3 (34.4)	-10.40 [-24.11, 3.31]	-0.32 [-0.77, 012]	.080
Aiarzaguena et al. (2007)	74<br><br>72	SF-36 Bodily pain	**Control:**<br>46.2 (25.2)<br><br>**Intervention:**<br>43.6 (24.4)			**Control:**<br>1.72 [b] [-2.69, 6.13] [a]<br>47.92 (19.35)*<br>**Intervention:**<br>11.41 [b] [6.98, 15.85] [a]<br>55.01 (19.1)*	7.09 [0.85, 13.33]	0.37 [0.04, 0.69]	.003

Mean and standard deviation unless specified otherwise. Items left blank were not provided within the paper.

*a = 95% CI, b = Mean difference, c = median, d = 25th – 75th percentiles, e = Interquartile range, * = mean and/or standard deviation calculated by author AB*

Table 2: Mental Functioning outcome data

Mean and standard deviation unless specified otherwise. Items left blank were not provided within the paper.

*a = 95% CI, b = Mean difference, c = median, d = 25th – 75th percentiles, e = Interquartile range, * = mean and/or standard deviation calculated by author 01*

Author	***N at 12 months***	***Measure***	***Baseline***	***3 month***	***6 month***	***12 month***	***Mean difference at 12 months between intervention and control [95% CI]***	***Standardised mean difference at 12 months [95% ci***	***P value at 12 months***
Rosendal et al. (2007)	283<br><br>299	SF-36 Mental Health Subscale	**Control:**<br>67.0 [64.4, 69.5][a]<br>67.0 (21.86)*<br><br>**Intervention:**<br>65.6 [63.4, 67.8] [a]<br>65.6 (19.36)*	**Control:**<br>0.8 [b] [-1.4, 3.1] [a]<br>67.8 (20.22)*<br><br>**Intervention:**<br>-0.7 [b] [-2.4, 1.0] [a]<br>64.9 (15.89)*		**Control:**<br>1.0 [b] [-1.9, 3.8] [a]<br>68 (24.38)*<br>**Intervention:**<br>0.4 [b] [-1.9, 2.6] [a]<br>66 (19.88)*	-2.00 {-5.63, 1.63]	-0.09 [-0.25, 0.07]	.760
	247<br><br>245	SF-36 Mental component summary	**Control:**<br>47.4 [45.8, 49.0] [a]<br>47.4 (12.89)<br><br>**Intervention:**<br>41.4 [44.9, 47.9] [a]<br>41.4 (12.05)	**Control:**<br>-0.1 [b] [-1.4, 1.2] [a]<br>47.3 (10.93)*<br><br>**Intervention:**<br>-0.6 [b] [-1.7, 0.5] [a]<br>40.8 (9.80)*		**Control:**<br>0.2 [b] [-1.4, 1.8] [a]<br>47.6 (12.83)*<br><br>**Intervention:**<br>-0.6 [b] [-1.8,0.6][a]<br>40.8 (9.55)*	-6.8 [-8.80,- 4.80]	-0.60 [-0.78, -0.42]	.420
Larisch et al. (2004)	34<br><br>44	SF-12 Mental	**Control:**<br>41.0 (10.3)<br><br>**Intervention:**<br>37.6 (9.6)	**Control:**<br>-1.6 [b] (2.3)<br><br>**Intervention:**<br>1.0 [b] (1.6)	**Control:**<br>-0.5 [b] (1.5)<br><br>Intervention:<br>1.5 [b] (2.2)	**Control:**<br>4.3 [b] (3.6)<br>45.3* (3.6)<br>**Intervention:**<br>2.2 [b] (5.2)<br>39.8*(5.2)	-5.50 [-7.46, -3.54]	-1.19 [1.68, -0.70]	.479
Schaefert et al. (2012)	108<br><br>143	SF-36 Mental component summary	**Control:**<br>40.38 (11.42)<br><br>**Intervention:**<br>41.55 (10.16)		**Control:**<br>42.21 (10.92)<br><br>**Intervention:**<br>45.63 (9.67)	**Control:**<br>42.09 (11.77)<br><br>**Intervention:**<br>46.59 (10.76)	4.50 [1.66, 7.34]	0.40 [0.15, 0.65]	.022
Aiarzaguena et al. (2007)	74<br><br>72	SF-36 Mental Health Subscale	**Control:**<br>50.3 (20.2)<br><br>**Intervention:**<br>50.1 (21.6)			**Control:**<br>5.63[b] [2.50, 8.76][a]<br>55.93 (13.67)*<br><br>**Intervention:**<br>10.27[b] [7.12, 13.42][a]<br>60.37 (13.65)*	4.44 [0.01, 8.87]	0.32 [-0.00, 0.65]	.063

Table 3: Somatisation outcome data

Author	*N at 12 months*	*Measure*	*Baseline*	*4 weeks*	*3 month*	*6 month*	*12 month*	*Mean difference at 12 months between intervention and control [95% CI]*	*Standardised mean difference at 12 months [95% CI]*	*P value at 12 months*
Rosendal et al. (2007)	284<br>304	SCL-SOM	**Control:**<br>2.3[c] (2.0-2.6)[d]<br>**Intervention:**<br>2.3[c] (1.9-2.6)[d]		**Control:**<br>-0.2[b] [-0.3, -0.2][a]<br>**Intervention:**<br>-0.2[b] [-0.2,-0.1][a]		**Control:**<br>-0.2[b] [-0.3, -0.2][a]<br>**Intervention:**<br>-0.2[b] [0.2, -0.1][a]			.230
Larisch et al. (2004)	34<br>44	SOM-7	**Control:**<br>12.3 (9.8)<br>**Intervention:**<br>14.8 (8.3)		**Control:**<br>1.5[b] (0.0)<br>13.8* (0.0)<br>**Intervention:**<br>-2.8[b] (-0.1)<br>12.0* (-0.1)	**Control:**<br>0.8[b] (1.2)<br>13.1* (1.2)<br>**Intervention:**<br>- 0.4 (1.9)[b]<br>14.4* (1.9)	**Control:**<br>1.6[b] (0.6)<br>13.9* (0.6)<br>**Intervention:**<br>-0.7[b] (3.1)<br>14.1* (3.1)	0.20 [-0.74, 1.14]	0.08 [-0.36, 0.53]	.192
Schaefert et al. (2012)	113<br>149	PHQ-15	**Control:**<br>12.66 (4.89)<br>**Intervention:**<br>12.56 (4.73)			**Control:**<br>11.42 (5.44)<br>**Intervention:**<br>9.47 (4.71)	**Control:**<br>10.57 (5.10)<br>**Intervention:**<br>9.55 (5.12)	-1.02 [-2.27, 0.23]	-0.20 [-0.44, 0.05]	.079
Rief et al. (2006)	114<br>85	SSI	**Control:**<br>6.8 (3.8)<br>**Intervention:**<br>8.0 (4.1)	**Control:**<br>5.2 (3.6)<br>**Intervention:**<br>6.3 (4.0)		**Control:**<br>5.9 (4.1)<br>**Intervention:**<br>5.9 (4.2)		0.00 [-1.17, 1.17]	0.00 [-0.28, 0.28]	
	114<br>85	SOMS symptom count	**Control:**<br>13.8 (8.2)<br>**Intervention:**<br>15.5 (8.2)	**Control:**<br>13.2 (7.5)<br>**Intervention:**<br>15.4 (8.2)		**Control:**<br>13.7 (8.2)<br>**Intervention:**<br>15.3 (9.5)		1.60 [-0.92, 4.12]	0.18 [-0.10. 0.46]	

Mean and standard deviation unless specified otherwise. Items left blank were not provided within the paper.

*a = 95% CI, b = Mean difference, c = median, d = 25th – 75th percentiles, e = Interquartile range, * = mean and/or standard deviation calculated by author 01*

Table 4: Anxiety outcome data

Author	***N at 12 months***	***Measure***	***Baseline***	***4 week***	***3 month***	***6 month***	***12 month***	***24 months***	***Mean difference at 12 months between intervention and control [95% CI]***	***Standardised mean difference at 12 months [95% CI]***	***P value at 12 months***
Rosendal et al. (2007)	285<br><br>306	SCL-8	**Control:**<br>1.9[c] (1.5-2.6)[d]<br><br>**Intervention:**<br>2.0[c] (1.5- 2.8)[d]		**Control:**<br>-0.2 [-0.3, -0.1][a]<br><br>**Intervention:**<br>-0.1 [-0.2, 0.0][a]		**Control:**<br>-0.2 [-0.3, -0.1][a]<br><br>**Intervention:**<br>-0.1 [-0.2, 0.0][a]				.320
Larisch et al. (2004)	34<br><br>44	HADS-A	**Control:**<br>7.7 (4.2)<br><br>**Intervention:**<br>9.3 (3.9)		**Control:**<br>0.3 b (0.2)<br>8.0* (0.2)<br><br>**Intervention:**<br>-1.1b (0.4)<br>8.2* (0.4)	**Control:**<br>-0.3 b (0.6)<br>7.4*(0.6)<br><br>Intervention:<br>-0.9 b (0.8)<br>8.8* (0.8)	**Control:**<br>-0.3 [b] (0.7)<br>7.4* (0.7)<br><br>**Intervention:**<br>-0.8[b] (0.7)<br>8.5* (0.7)		1.10 [0.79, 1.41]	1.56 [1.04, 2.07]	.419
Schaefert et al. (2012)	113<br><br>149	Whiteley-7	**Control:**<br>10.37 (6.24)<br><br>**Intervention:**<br>10.86 (6.61)			**Control:**<br>8.60 (6.15)<br><br>**Intervention:**<br>7.75 (6.26)	**Control:**<br>8.57 (6.93)<br><br>**Intervention:**<br>7.66 (6.60)		-0.91 [-2.57, 0.75]	-0.13 [-0.38, 0.11]	.061
Morriss et al. (2007)	75<br><br>66	Caseness (n) and %	**Control:**<br>46 (61%)<br><br>**Intervention:**<br>40 (64%)		**Control:**<br>27 (36%)<br><br>**Intervention:**<br>31 (47%)						.101

Toft et al. (2010)		SCL-8	**Control:**<br>16c (12-21)[e]<br>**Intervention:**<br>16c (12-21)[e]								
	125<br>154	Whiteley-7	**Control:**<br>13c (10-17)[e]<br>**Intervention:**<br>13c (10-17)[e]						3.0 [0.8, 3.9][x]		.001
Alamo, Moral & de Torres (2002)	33<br>48	GHQ-anxiety	**Control:**<br>5.2 (2.9)<br>**Intervention:**<br>6.2 (2.4)			**Control:**<br>5.4 (2.9)<br>**Intervention:**<br>5.4 (2.7)	**Control:**<br>5.4 (2.8)<br>**Intervention:**<br>4.6 (4.8)		-0.80 [-2.46, 0.86]	-0.19 [-0.64, 0.25]	.040
Rief et al. (2006)	114<br>85	BAI	**Control:**<br>11.8 (10.0)<br>**Intervention:**<br>14.4 (10.3)	**Control:**<br>10.2 (8.6)<br>**Intervention:**<br>14.0 (11.1)		**Control:**<br>11.5 (9.2)<br>**Intervention:**<br>11.8 (10.6)			0.30 [-2.52, 3.12]	0.03 [-0.25, 0.31]	
	114<br>85	WI	**Control:**<br>5.4 (3.1)<br>**Intervention:**<br>6.2 (2.9)	**Control:**<br>4.7 (3.2)<br>**Intervention:**<br>5.2 (3.1)		**Control:**<br>4.6 (3.1)<br>**Intervention:**<br>5.0 (3.3)			0.40 [-0.50, 1.30]	0.13 [-0.16, 0.41]	

Mean and standard deviation unless specified otherwise. Items left blank were not provided within the paper.

*a = 95% CI, b = Mean difference, c = median, d = 25th – 75th percentiles, e = Interquartile range, * = mean and/or standard deviation calculated by author 01 x = mean difference provided within original data*

Table 5: Depression outcome data

Author	***N at 12 months***	***Measure***	***Baseline***	***4 weeks***	***3 month***	***6 month***	***12 month***	***Mean difference at 12 months between intervention and control [95% CI]***	***Standardised mean difference at 12 months [95% CI]***	***P value at 12 months***
Rosendal et al. (2007)	285<br><br>306	SCL-8	**Control:**<br>1.9[c] (1.5-2.6)[d]<br><br>**Intervention:**<br>2.0[c] (1.5-2.8) [d]		**Control:**<br>-0.2[b] [-0.3, -0.1][a]<br><br>**Intervention:**<br>-0.1 [-0.2, 0.0]		**Control:**<br>-0.2[b] [-0.3, -0.1][a]<br><br>**Intervention:**<br>-0.1[b] [-0.2, 0.0] [a]			.320
Larisch et al. (2004)	34<br><br>44	HADS-D	**Control:**<br>6.3 (3.5)<br><br>**Intervention:**<br>7.9 (4.5)		**Control:**<br>0.5[b] (1.1)<br>6.8 (1.1)*<br><br>**Intervention:**<br>-1.2 [b] (-0.3)<br>6.7 (-0.3)*	**Control:**<br>-0.4 [b] (0.9)<br>5.9 (0.9)*<br><br>Intervention:<br>-0.7 [b] (0.1)<br>7.2 (0.1)*	**Control:**<br>-0.2 [b] (0.7)<br>6.1 (0.7)*<br><br>**Intervention:**<br>-0.8 [b] (0.0)<br>7.1 (0.0)*	1.00 [0.76, 1.24]	2.15 [1.58, 2.71]	.467
Schaefert et al. (2012)	112<br><br>149	PHQ-9	**Control:**<br>9.76 (5.54)<br><br>**Intervention:**<br>8.89 (5.11)			**Control:**<br>8.12 (4.92)<br><br>**Intervention:**<br>6.68 (4.82)	**Control:**<br>7.98 (5.25)<br><br>**Intervention:**<br>6.29 (4.58)	-1.69 [-2.91, -0.47]	-0.35 [-0.59, -0.10]	.111
Morriss et al. (2007)	75<br><br>66	Caseness and %	**Control:**<br>46 (61%)<br><br>**Intervention:**<br>40 (64%)		**Control:**<br>21 (28%)<br><br>**Intervention:**<br>18 (27%)					.873
Toft et al. (2010)	125<br><br>154	SCL-8	**Control:**<br>16[c] (12-21)[e]<br>**Intervention:**<br>16[c] (12-21)[e]							

Alamo, Moral & de Torres (2002)	33<br><br>48	GHQ-depression	**Control:**<br>3.6 (2.5)<br><br>**Intervention:**<br>3.7 (2.5)			**Control:**<br>3.9 (2.4)<br><br>**Intervention:**<br>3.6 (2.5)	**Control:**<br>4.0 (2.1)<br><br>**Intervention:**<br>3.2 (2.6)	-0.80 [-1.83, 0.23]	-0.33 [-0.78, 0.12]	.330
Rief et al. (2006)	114<br><br>85	BDI	**Control:**<br>12.5 (8.4)<br><br>**Intervention:**<br>13.9 (9.1)	**Control:**<br>12.2 (9.3)<br><br>**Intervention:**<br>13.6 (9.5)		**Control:**<br>11.8 (8.1)<br><br>**Intervention:**<br>11.8 (9.5)		0.00 [- 2.51, 2.51]	0.00 [-0.28, 0.28]	

Mean and standard deviation unless specified otherwise. Items left blank were not provided within the paper.

*a = 95% CI, b = Mean difference, c = median, d = 25th – 75th percentiles, e = Interquartile range, * = mean and/or standard deviation calculated by author 01*

Table 6: Physical functioning outcome data

Author	***N at 12 months***	***Measure***	***Baseline***	***3 month***	***6 month***	***12 month***	***24 month***	***Mean difference at 12 months between intervention and control [95% CI]***	***Standardised mean difference at 12 months [95% CI]***	***P value at 12 months***

Rosendal et al. (2007)	284<br><br>288	SF-36	**Control:**<br>84.2[c] (65.0-95.0)[d]<br><br>**Intervention:**<br>80.0[c] (61.1-95.0)[d]	**Control:**<br>-0.3[b] [-2.2, 1.6][a]<br><br>**Intervention:**<br>**-0.1**[b] [-2.3, 2.2]		**Control:**<br>0.8[b] [-0.9, 2.6][a]<br><br>**Intervention:**<br>0.5[b] [-1.7, 2.8][a]				.890
Larisch et al. (2004)	34<br><br>44	SF-12	**Control:**<br>43.0 (11.0)<br><br>**Intervention:**<br>41.4 (8.2)	**Control:**<br>-0.8 (-1.2)<br>42.2 (-1.2)*<br><br>**Intervention:**<br>2.8 (0.7)<br>43.2 (0.7)*	**Control:**<br>1.2 (-0.4)<br>44.2 (-0.4)*<br><br>**Intervention:**<br>2.7 (1.6)<br>43.1 (1.6)*	**Control:**<br>0.5 (-0.7)<br>43.5 (-0.7)*<br><br>**Intervention:**<br>3.8 (2.0)<br>45.2 (2.0)*		1.70 [1.06, 2.34]	-3.08 [-3.75, -2.41]	.069
Aiaraguena et al. (2007)	74<br><br>72	SF-36	**Control:**<br>70.5 (25.1)<br><br>**Intervention:**<br>73.2 (23.2)			**Control:**<br>2.56[a] (1.15, 3.97)[b]<br>73.06 (6.20)*<br><br>**Intervention:**<br>5.23[b] (3.8-6.67)[a]<br>78.43 (6.20)*		5.37 [3.36, 7.38]	0.86 [0.52, 1.20]	.012
Schaefert et al. (2012)	108<br><br>143	SF-36	**Control:**<br>42.05 (8.88)<br><br>**Intervention:**<br>43.16 (9.09)		**Control:**<br>43.19 (9.99)<br><br>**Intervention:**<br>44.49 (8.49)	**Control:**<br>44.14 (9.68)<br><br>**Intervention:**<br>44.56 (9.61)		0.42 [-1.99, 2.83]	0.04 [-0.21, 0.29]	.674
Toft et al. (2010)	111<br><br>138	SF-36	**Control:**<br>90.0[c] (75.0-100.0)[e]<br><br>**Intervention:**<br>85.0[c] (60.0-95.0)[e]							
Alamo, Moral & de Torres (2002)	33<br><br>48	NHP-physical mobility	**Control:**<br>29.2 (19.4)<br><br>**Intervention:**<br>22.7 (17.9)		**Control:**<br>30.0 (19.8)<br><br>**Intervention:**<br>22.6 (18.0)	**Control:**<br>32.2 (21.5)<br><br>**Intervention:**<br>20.1 (16.3)		-12.10 [-20.76, -3.44]	-0.64 [-1.10, -0.19]	.100

Mean and standard deviation unless specified otherwise. Items left blank were not provided within the paper.

*a = 95% CI, b = Mean difference, c = median, d = 25th – 75th percentiles, e = Interquartile range, * = mean and/or standard deviation calculated by author 01*

Table 7: Clinician outcome measures reported by Rosendal et al. (2005)

		Baseline (All 41 participants)					*12 months*			*Mean difference*	*P value*
Questionnaire item	**Related outcome**	**Mean**	**SD**	**95% CI**	**n**	**Allocation**	**Mean (difference)**	**SD**	**95% CI**		
Item 3. I often feel unsure of what to do	Confidence	3.4			22	Intervention	-1.0	1.19*	[-1.5, -0.5]	-0.90 [-1,72, -0.08]	.019
					17	Control	-0.1	1.36*	[-0.8, 0.5]		
Item 16. I feel comfortable in dealing with somatising patients	Confidence	4.5	2.44*	[3.7, 5.2]	22	Intervention	2.2	2.39*	[1.2, 3.2]	2.50 [0.86, 4.14]	.002
					17	Control	-0.3	2.73*	[-1.6, 1.0]		
Item 4. I enjoy working with these patients	MUS beliefs	3.0			22	Intervention	0.9	1.07*	[0.4, 1.3]	1.10 [0.27, 1.93]	.008
					17	Control	-0.2	1.47*	[-0.9,0.5]		
Item 17. Somatisation reflects a characteristic response in patients which is not amenable to change	MUS beliefs	3.7	2.28*	[3.0, 4.4]	22	Intervention	-0.8	2.75*	[-2.0, 0.3]	-0.60 [-2.19, 0.99]	.441
					17	Control	-0.2	2.31*	[-1.3, 0.9]		

Mean and standard deviation unless specified otherwise. Items left blank were not provided within the paper.

*a = 95% CI, b = Mean difference, c = median, d = 25th – 75th percentiles, e = Interquartile range, * = mean and/or standard deviation calculated by author 01*
