# Supplementary material for: Communication interventions for medically unexplained symptom conditions in general practice: A systematic review and meta-analysis of randomised controlled trials
Source: PLoS One. 2022 Nov 14;17(11):e0277538. doi: 10.1371/journal.pone.0277538 (PMC9662736; doi:10.1371/journal.pone.0277538)
Supplement: S8 Table — (PDF) [file pone.0277538.s008.pdf]

Supplementary material - Appendix 1: Example search strategy

Search terms

1. Somatoform disorders
2. Somatoform
3. Psychosomatic
4. Medically unexplained symptoms
5. MUS
6. MUPS
7. Medically unexplained physical symptoms
8. Fibromyalgia
9. Vulvodynia
10. Irritable Bowel Syndrome
11. IBS
12. Chronic widespread pain
13. Chronic Fatigue syndrome
14. Tension type headache
15. 1 OR 2 OR 3 OR 4 OR 5 OR 6 OR 7 OR 8 OR 9 OR 10 OR 11 OR 12 OR 13 OR 14
16. Randomised controlled trial
17. Randomized controlled trial
18. Trial
19. Controlled clinical trial
20. 16 OR 17 OR 18 OR 19
21. GP*
22. General practitioner*

23. Primary care
24. Health professional
25. Doctor*
26. Physician*
27. 21 or 22 or 23 or 24 or 25 or 26
28. Patient centred care
29. Person centred care
30. Communication
31. 28 or 29 or 30
32. 15 AND 20
33. 32 AND 31
