# Supplementary material for: Communication interventions for medically unexplained symptom conditions in general practice: A systematic review and meta-analysis of randomised controlled trials
Source: PLoS One. 2022 Nov 14;17(11):e0277538. doi: 10.1371/journal.pone.0277538 (PMC9662736; doi:10.1371/journal.pone.0277538)
Supplement: S9 Table — (PDF) [file pone.0277538.s009.pdf]

Supporting material - Appendix 2: Summary of the assessed communication interventions

**Author**	**Intervention type**	**Control type**	**Overview of intervention aim(s)**	**Number and length of sessions**	**Content of intervention**	**Who provided the training?**	***Author opinion* – does the trial provide sufficient detail to replicate the intervention?**
**<u>Reattribution training</u>**							
**Rosendal et al. (2005) ***	Multifaceted educational programme based upon the TERM model (reattribution)	Clinicians were informed about the definitions of somatization	- To mediate evidence about somatization<br>- Give GPs general interview techniques and specific principles for somatisation using cognitive, behavioral and administrative techniques<br>- Change attitudes towards somatizing patients	2 day residential course<br><br>3 x 2 hour follow-up meetings<br><br>1 x 3 month booster meeting<br><br>1 x 6 month outreach visit<br><br>1 x posted information after 9 months	2 day residential = Theory and evidence about somatization, treatment model, introduction to exercises, video training, group discussion, micro skills training with actors, role-play.<br><br>Follow-up meetings = Group meetings and video supervision<br><br>Booster meeting = Small group discussion<br><br>Outreach visit = Supervisors visited practices to reinforce learning<br><br>Posted information = Letter describing overall experiences from outreach visits	'Supervisors'. No further detail provided	No
**Rosendal et al. (2007) ****Associated paper***						'	
**Morriss et al. (2007)**	Reattribution training	Treatment as usual	Aimed to teach clinicians how to provide a 3 stage psychological explanation to patients for their MUS – psychosocial, physiological or temporal mechanism	3 x 2 hour training session	Teaching clinicians 1.) How to make the patient feel understood, 2.) To broaden the agenda, making the link between physical symptoms and psychosocial or physiological issues, and 3.) To negotiate further treatment with the patient.<br>Achieved using videotaped training	Three nurses and a psychologist ('health facilitators') were trained by a specialist	No

					materials, information on the reattribution model, role play and videotaped feedback of consultations		
**Toft et al. (2010)**	The Extended Reattribution and Management Model	Informed about the definitions of functional somatic syndrome, somatic disorder and the contents of the questionnaires	To provide GPs with theoretical knowledge and practical skills for the diagnosis and treatment of MUS through 5 steps: 1.) Understanding, 2.) Exploring the physician's expertise and acknowledgement, 3.) Negotiating a new model of understanding, 4.) Negotiating further treatment, 5.) How to manage chronic patients	2 day residential course<br><br>3 x 2 hour weekly evening sessions<br><br>1 x 3 month booster meeting<br><br>1 x supervisor outreach visit at 6 months	- Videotaped consultations with patients<br>- Interview techniques<br><br>Authors provide no information on how training was divided up between sessions.	Not given	No, although state that the manual and training are available on request
**Morriss et al. (2006)**	Reattribution training	Informed about the definitions of somatization and somatoform disorder	Training included developing the following actions in the GP: 1.) recognizing the patient's problems, 2.) explaining how physical and psychological symptoms are linked, 3.) using a patient-centered approach, 4.) reducing the number of referrals, drugs and tests the GP orders, 5.) increasing mental health treatment for the patient	3 x 2 hour training sessions	- Videotaped training material	4 health facilitators (3 mental health facilitators and 1 primary care professional)	No
**Larisch et al. (2004)**	Psychosocial intervention based upon the Reattribution Model	Routine primary care	Training focused on the specific skills necessary for the management of somatizing patients. Training conveyed knowledge by: 1.) taking the patient's complaints seriously, 2.) conduct the consultation without questioning the patient's experience, explain symptoms and explain them in acceptable language, explore stressors and explain the possible pathophysiology.	1 x 8 hour class<br><br>1 x 4 hour evening class	- Video feedback<br>- Role play<br>- Video demonstrations<br>- Case discussion<br>- Modelling of behavior<br>- Video evaluations<br><br>No information was provided on how training was divided up between sessions.	Authors	No, although reference to a treatment manual was provided.
**<u>Patient Centered Approach</u>**							

**Alamo, Moral & de Torres (2002)**	Patient centered approach	Treatment as usual	Aimed to train clinicians in the application of the communication skills needed to undertake a patient centered approach	No information	No information given on the educational content of the training.<br><br>Features of the patient centered approach covered in the training included: listening to the patient, exploring the patient's condition, symptoms and expectations, being supportive and empathetic, finding agreement and common ground, and naming the processes.	.No information	No
**<u>Other</u>**							
**Aiarzaguena et al. (2007)**	Specific standardized communication technique for somatization, emphasizing a physical diagnosis for presenting symptoms	Goldberg's Reattribution technique	GPs were trained to: 1.) explain the patients MUS in a physical and tangible manner, 2.) explore psychosocial aspects, 3.) attribute physical symptoms to irrational thoughts. Designed to reinforce, legitimize and reinforce patient's experiences	5 x 4 hour sessions delivered over 2.5 days	- Standardized communication techniques<br>- Short theoretical lecture<br>- Small group discussion<br>- Role play	No information	No
**Schaefert et al. (2012)**	Collaborative group intervention (interpersonal approach of psychodynamically based therapy) and enhanced medical care training	Enhanced medical care training only	Training goals were 1.) To build a better understanding of MUS, 2.) Able to empower patients to achieve mental and physical stabilization and coping skills, 3.) Strength the patient's self-perception, 4.) Create an awareness of the interaction of symptoms with intra- and inter-personal conflicts	10 x 90 minute weekly group sessions<br><br>2 x booster sessions at 3 and 9 months	Group sessions = Information about MUS, cohesion & self-disclosure, focusing, resource activation, verbalization and mentalization of bodily complaints, relaxation training, graded physical activation, understanding stress triggers, appraisal processes, illness beliefs, affect perception, tolerance, coping strategies, interpersonal learning, dealing with relapse, and planning.<br><br>Booster sessions = self-efficacy	3 psychosomatic specialists and the principle investigator	No, although reference to a treatment manual was provided and a synopsis was given within the paper.

**Rief et al. (2006)**	A training packing on managing patients with unexplained physical symptoms, including how to communicate with them.	No training	To help GP's use management strategies in interaction with MUS patients.	1 day session	Presentation and discussion of management guidelines, and, if necessary, role-play. The management guidelines were a synthesis of those used in other studies, further research results, and our own experience. Topics included: how to communicate with patients, when to start and when to stop medical examinations, how to handle the organic health beliefs of the patients, their need for reassurance, and their avoidance of physical activity.	No information.	No
