# Supplementary material for: Communication interventions for medically unexplained symptom conditions in general practice: A systematic review and meta-analysis of randomised controlled trials
Source: PLoS One. 2022 Nov 14;17(11):e0277538. doi: 10.1371/journal.pone.0277538 (PMC9662736; doi:10.1371/journal.pone.0277538)
Supplement: S10 Table — (PDF) [file pone.0277538.s010.pdf]

Supplementary material – Appendix 3 – Patient characteristics

Author	N	Mean (SD) age in years	Gender breakdown	MUS condition	Occupation	Education	Marital status
Rosendal et al. * (2005)							
***Rosendal et al. (2007)* *Associated paper**	911	**Intervention:** 41.6 [39.6, 43.6][b] **Control:** 42.2 [40.9, 43.4][b]	**Intervention:** 192 (37.9%) male **Control:** 127 (31.4%) male	Undistinguishable mixture of MUS conditions	**Intervention:** 273 (57.6%) employed **Control:** 240 (61.5%) employed	**Intervention:** 295 (60.7%) accomplished primary school only **Control:** 231 (57.8%) accomplished primary school only	**Intervention:** 337 (67.8%) living with partner or spouse **Control:** 279 (69.4%) living with partner or spouse
Morriss et al. (2007)	141	**Intervention:** 53.9 (11.9) **Control:** 48.9 (13.8)	**Intervention:** 54 (72%) female **Control:** 45 (68%) female	Undistinguishable mixture of MUS conditions			
Alamo, Moral & de Torres (2002)	110	**Intervention:** 39.2 (7.6) **Control:** 42.3 (10.0)	**Overall:** 107 females (97.3%) and 3 males (2.7%)	Fibromyalgia or chronic musculoskeletal pain	Housewives = 60 (54.5%) Other = 50 (45.5%)		Married: 97 (88.2%) Other: 13 (11.8%)
Aiarzaguena et al. (2007)	156	**Intervention:** 47.2 (10.6%) **Control:** 46.9 (11.7%)	**Intervention:** 61 (82.4%) female **Control:** 62 (79.5%) female	Undistinguishable mixture of MUS conditions			
Larisch et al. (2004)	127	**Intervention:** 47.7 (11.8) **Control:** 42.5 (12.2)	**Intervention:** 20 male (27.4%), 53 female (72.6%) **Control:** 15 male (27.8%), 39 female (72.2%)	Undistinguishable mixture of MUS conditions	**Intervention:** Employed: 41 (65.1%) Unemployed: 8 (12.7%) Disabled: 2 (3.2%) Retired: 2 (3.2%) Other: 10 (15.9%) **Control:** Employed: 29 (59.2%) Unemployed:3 (6.1%) Disabled: 5 (10.2%) Retired: 4 3.2% Other: 8 (16.3%)	**Intervention:** Basic school: 37 (53.6%) High school: 18 (26.1%) A-levels: 13 (18.8%) School drop-out: 1 (1.4%) **Control:** Basic school: 26 (49.1%) High school: 19 (35.8%) A-levels: 7 (13.2%) School drop-out: 1 (1.9%)	**Intervention:** Single: 13 (18.1%) Married/partner: 54 (75%) Widowed:0 (0%) Divorced: 5 (6.9%) **Control**: Single: 4 (7.8%) Married/partner: 44 (86.3%) Widowed: 1 (2%) Divorced: 2 (3.9%)
Schaefert et al. (2012)	328	**Intervention:** 50.8 (12.0)	**Intervention:** 128 (75.3%) female	Undistinguishable mixture of MUS conditions	**Intervention:** Employed: 84 (50%)	**Intervention:** ISCED secondary or	**Intervention:**

		**Control:**<br>46.6 (12.9)	**Control:**<br>100 (74.6%) female		Disabled: 21 (12.4%)<br>Retired: 30 (17.8%)<br><br>**Control:**<br>Employed: 75 (56%)<br>Disabled: 15 (11.3%)<br>Retired: 16 (12%)	lower: 135 (81.8%)<br><br>**Control:**<br>100 (80%)	Living with partner: 125 (74.9%)<br><br>**Control:**<br>Living with partner: 68 (51.9%)
Toft et al. (2010)	350	**Intervention:**<br>43.4[a]<br>**Control:**<br>39.0[a]	**Intervention:**<br>46 males (25%), 149 females (76%)<br><br>**Control:**<br>47 males (30%), 108 females (70%)	Undistinguishable mixture of MUS conditions/somatic disorder	**Intervention:**<br>Employed: 101 (51.8%),<br>Unemployed/student: 74 (38.0%)<br>Other: 20 (10.2%)<br><br>**Control:**<br>Employed: 72 (46.5%),<br>Unemployed/student: 72 (46.5%)<br>Other: 11 (7.0%)	**Intervention:**<br>Basic school: 96 (49.3%)<br>Further education: 81 (41.5%)<br>Other: 18 (9.2%)<br><br>**Control:**<br>Basic school: 78 (50.3%)<br>Further education: 68 (43.9%)<br>Other: 9 (5.8%)	
Morriss et al. (2006)							
Rief et al. (2006)	289	**Overall mean age:**<br>50.6	**Overall gender breakdown:**<br>65% female (188), 35% male (101)	Undistinguishable mixture of MUS conditions			

Mean and standard deviation unless specified otherwise. Items left blank were not provided within the paper.
a = median
b = 95% CI
